# Risk for Esophageal Cancer Based on Lifestyle Factors–Smoking, Alcohol Consumption, and Body Mass Index: Insight from a South Korean Population Study in a Low-Incidence Area

**DOI:** 10.3390/jcm12227086

**Published:** 2023-11-14

**Authors:** Mi Jung Kwon, Ho Suk Kang, Hyo Geun Choi, Joo-Hee Kim, Ji Hee Kim, Woo Jin Bang, Sung Kwang Hong, Nan Young Kim, Sangkyoon Hong, Hong Kyu Lee

**Affiliations:** 1 Department of Pathology, Hallym University Sacred Heart Hospital, Hallym University College of Medicine, Anyang 14068, Republic of Korea; mulank99@hallym.or.kr; 2 Division of Gastroenterology, Department of Internal Medicine, Hallym University Sacred Heart Hospital, Hallym University College of Medicine, Anyang 14068, Republic of Korea; hskang76@hallym.or.kr; 3 Suseo Seoul E.N.T. Clinic and MD Analytics, 10, Bamgogae-ro 1-gil, Gangnam-gu, Seoul 06349, Republic of Korea; mdanalytics@naver.com; 4 Division of Pulmonary, Allergy, and Critical Care Medicine, Department of Medicine, Hallym University Sacred Heart Hospital, Hallym University College of Medicine, Anyang 14068, Republic of Korea; luxjhee@hallym.or.kr; 5 Department of Neurosurgery, Hallym University Sacred Heart Hospital, Hallym University College of Medicine, Anyang 14068, Republic of Korea; kimjihee@hallym.or.kr; 6 Department of Urology, Hallym University Sacred Heart Hospital, Hallym University College of Medicine, Anyang 14068, Republic of Korea; yybbang@hallym.or.kr; 7 Department of Otorhinolaryngology-Head & Neck Surgery, Hallym University College of Medicine, Anyang 14068, Republic of Korea; yerami@hallym.or.kr; 8 Hallym Institute of Translational Genomics and Bioinformatics, Hallym University Medical Center, Anyang 14068, Republic of Korea; honeyny@hallym.or.kr (N.Y.K.); kyoons@hallym.or.kr (S.H.); 9 Department of Thoracic and Cardiovascular Surgery, Hallym University Sacred Heart Hospital, Hallym University College of Medicine, Anyang 14068, Republic of Korea

**Keywords:** esophageal cancer, cancer risk, smoking, alcohol consumption, body mass index, national health screening cohort research database

## Abstract

Esophageal cancer constitutes a global public health challenge. However, South Korean population-specific information on the association of lifestyle (smoking, alcohol consumption, and obesity status) with esophageal cancer risk is sparse. This nested case–control study analyzed the Korean national health screening cohort data (2002–2019) of 1114 patients with esophageal cancer and 4456 controls (1:4 propensity-score matched for sex, age, income, and residential region). Conditional and unconditional logistic regression analyses, after adjustment for multiple covariates, determined the effects of lifestyle factors on esophageal cancer risk. Smoking and alcohol consumption increased the esophageal cancer risk (adjusted odds ratio [95% confidence interval]: 1.37 [1.15–1.63] and 1.89 [1.60–2.23], respectively). Overweight (body mass index [BMI] ≥ 23 to <25 kg/m^2^), obese I (BMI ≥ 25 to <30 kg/m^2^), or obese II (BMI ≥ 30 kg/m^2^) categories had reduced odds of esophageal cancer (0.76 [0.62–0.92], 0.59 [0.48–0.72], and 0.47 [0.26–0.85], respectively). In the subgroup analyses, the association of incident esophageal cancer with smoking and alcohol consumption persisted, particularly in men or those aged ≥55 years, whereas higher BMI scores remained consistently associated with a reduced esophageal cancer likelihood across all age groups, in both sexes, and alcohol users or current smokers. Underweight current smokers exhibited a higher propensity for esophageal cancer. In conclusion, smoking and alcohol drinking may potentially increase the risk, whereas weight maintenance, with BMI ≥ 23 kg/m^2^, may potentially decrease the risk, for esophageal cancer in the South Korean population. Lifestyle modification in the specific subgroups may be a potential strategy for preventing esophageal cancer.

## 1. Introduction

Esophageal carcinomas constitute a significant global health challenge, ranking seventh in prevalence and sixth in cancer-related mortality [1]. These malignancies are highly aggressive and challenging to treat, with a low 5-year survival rate of 10–30% [1,2], with poor outcomes that are partly attributable to an advanced stage at diagnosis, as well as the inherent resistance of esophageal cancers to systemic therapy because of histological, molecular, and etiological heterogeneity [3]. In recent decades, the occurrence of esophageal cancer in South Korea has decreased, making it the fifteenth most prevalent cancer, constituting 1.0% of all cancer cases [4,5]. However, esophageal cancer remains a substantial public health concern, ranking within the top 10 leading causes of cancer-related fatalities in South Korea, with a notable sex disparity (10.2:1 male-to-female ratio) and a persistently high fatality rate of 65%, as compared with that of 30% for all cancer patients [5,6]. Esophageal cancer is most commonly found in East Asia, followed by Southern Africa, East Africa, and Northern Europe, for both sexes [1,2]. The incidence of esophageal cancer in South Korea (2.6) [4] appears to be lower than that in other East Asian countries such as Japan (2.8–5.7) [7,8], China (12.7) [9], and Taiwan (23.83) [10], with potential differences in risk factor profiles suggested as a contributing factor, although the precise reasons remain unclear.

Esophageal cancer may arise in part due to various environmental lifestyle factors including smoking, alcohol consumption, obesity, overweight, environmental exposures, dietary habits, and nutrition [11,12,13,14], which have garnered significant attention as modifiable risk factors. A recent meta-analysis of six studies emphasized the significant link between lifestyle factors such as smoking and alcohol consumption and esophageal cancer among Asians, particularly those of Chinese, Japanese, and Taiwanese descent [11]. This association was particularly pronounced in regions where squamous cell carcinoma histology prevails and wherein esophageal cancer has a high incidence in the populations [11]. However, the association within a region of relatively low incidence, such as South Korea, has been infrequently reported [15].

Obesity is a global health issue, with obesity-related health burdens increasing worldwide [16]. In South Korea, rapid economic development has triggered dietary shifts toward westernization and has substantially increased obesity rates over a relatively brief period, with up to 40% of the population categorized as obese (defined as a body mass index, or BMI ≥ 25 kg/m^2^) [17]. Notably, South Koreans, while generally leaner compared to Western populations, exhibit a higher susceptibility to obesity-related diseases at a given BMI level [16,17]. Prior meta-analyses have revealed that while an increase in BMI appears to elevate the risk of esophageal cancer in European and North American populations, it appears to have the opposite effect by reducing this risk among Asians [18,19]. Nonetheless, a more recent meta-analysis conducted in 2019, which incorporated underweight as a newly added category, found a consistent association between being underweight and an elevated risk of esophageal cancer, irrespective of ethnic background [20]. This necessitates a clear assessment of the association between BMI and overall esophageal cancer risk, with a focus on the South Korean population. A population-based study conducted in South Korea investigated the joint effects of low BMI and alcohol intake on incident esophageal cancers, while stratifying the data by age, sex, smoking, and insufficient BMI categories [21]. However, the interpretation of the results might be limited due to the uneven baseline characteristics between the study and control cohorts. Such heterogeneity in baseline characteristics across cohorts can distort the generalizability of findings [22]. Despite the increasing aging population and evolving lifestyles in South Korea over several decades owing to a rapid transition to a developed country [23], there is limited information regarding this issue in the Korean population.

In South Korea, a substantial portion of the population, including 66.0% of men and 43.9% of women aged ≥30 years [15], exhibits multiple concurrent risk factors, such as active smoking and high-risk alcohol consumption or active smoking and obesity [24]. These findings underscore the importance of prioritizing integrated risk factor management in disease prevention and management strategies, rather than addressing individual risk factors in isolation [25]. Hence, there is a need for additional research to explore the separate and combined impacts of alcohol consumption, smoking, and obesity status on the onset of esophageal cancer and to determine their respective contributions to the disease risk.

We hypothesized that the influence of lifestyle factors, encompassing weight status, smoking habits, and alcohol consumption, on the susceptibility to esophageal cancer might exhibit variations contingent on individual characteristics such as sex, age, and distinct patterns of smoking, alcohol use, and obesity status within the South Korean population. By utilizing a validated Korean health screening database and adjusting for relevant confounders, we simultaneously assessed the influence of these lifestyle factors on the occurrence of esophageal cancer in adults, while considering detailed subgroup analyses of smoking, alcohol drinking, and obesity status.

## 2. Materials and Methods

The research obtained ethical approval from the Hallym University ethics committee (2019-10-023), and written informed consent was waived by the Institutional Review Board. This study utilized data from the Korean National Health Insurance Service-Health Screening Cohort (KNHIS-HSC) from 2002 to 2019, which provides population-based research data in South Korea. The National Health Insurance Service (NHIS) covers over 98% of the South Korean population due to a mandatory nationwide health insurance policy, ensuring extensive representation. To protect privacy, beneficiary identification codes were deidentified for complete anonymity. South Korean residents aged ≥40 years are required to undergo biennial health screening as part of the NHIS program, occurring every 2 years. The NHIS-HSC cohort, established in 2015, originated from a sample cohort selected from participants in health screenings conducted in 2002 and 2003 by the NHIS in South Korea [26]. This cohort included individuals aged 40–79 years in 2002 and was tracked until 2019, comprising 514,866 health screening participants, representing a 10% random subset of all participants from 2002 and 2003, equivalent to approximately 3% of the South Korean adult population. Regarding follow-ups, 31.6% of participants were biennially monitored until 2013 and 93.6% underwent screenings at least once post-baseline. The NHIS remains committed to maintaining and regularly updating this cohort [26].

In a retrospective nested case–control cohort study, we investigated the impact of smoking, alcohol consumption, and obesity on the development of esophageal cancer within two distinct groups: those with esophageal cancer (*n* = 1169) identified using ICD-10 code C15 and a comparison group (*n* = 513,697) from a database of 514,866 individuals aged ≥40 years with 895,300,177 medical claim codes recorded from 2002 to 2019. To ensure incident cases, we excluded those diagnosed in 2002 (*n* = 47) and established the index date for esophageal cancer patients as the date of the ICD-10 code assignment (C15) in the health insurance claims dataset. We also excluded esophageal cancer participants without prior BMI records (*n* = 8) and control participants with a single esophageal cancer diagnosis (ICD-10 code C15) but no related treatments (*n* = 381).

**Figure 1:**
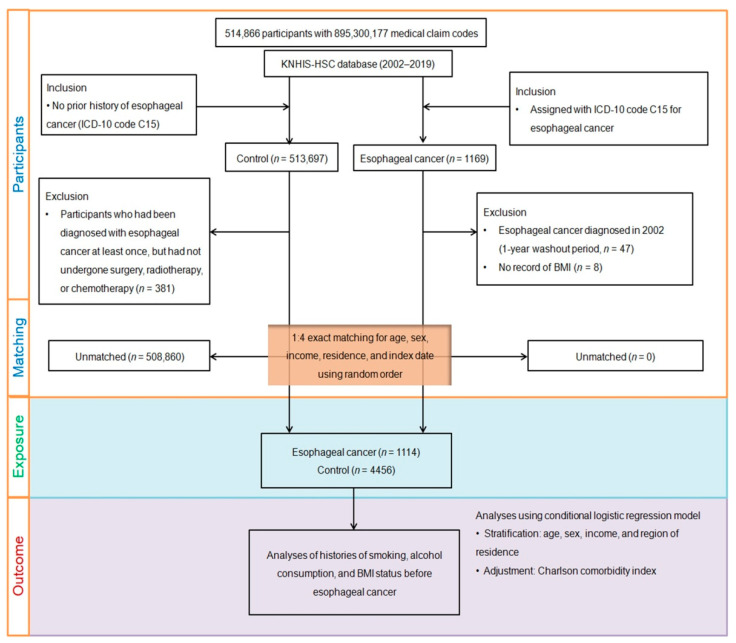
The participant selection process employed in this study is depicted in the following schematic illustration. Initially, a total of 514,866 participants were included. From this pool, 1114 participants diagnosed with esophageal cancer were matched in a 1:4 ratio with 4456 control participants based on age, sex, income, and region of residence. BMI, body mass index; ICD-10; International Classification of Diseases, 10th Revision; KNHIS-HSC, Korean National Health Insurance Service-Health Screening Cohort.

Propensity score matching was utilized to establish a more equitable distribution of baseline characteristics between individuals afflicted with esophageal cancer and those in the control group. It considered variables such as age, sex, income, and residence and employed random clustered sampling to minimize selection biases. Control participants were aligned with their esophageal cancer counterparts in terms of index dates, resulting in identical timing for each matched pair. This meticulous matching process excluded 508,860 control members who could not be matched, ultimately resulting in 1114 individuals with esophageal cancer being successfully paired with 4456 control participants at a 1:4 ratio for comparison (Figure 1).

### 2.1. Exposure (Smoking, Alcohol Consumption, and Obesity)

To gather comprehensive information on smoking history, alcohol consumption, and BMI scores before the diagnosis of esophageal cancer, a retrospective search was conducted within the cohort groups. As the medical examination for the health screening program provided by the NHIS occurs every 2 years [26], the BMI data were collected from the 2 most recent years before the index date in this study. Smoking status was categorized as non-smoker, ex-smoker, or current smoker [27]. Alcohol consumption was assessed based on frequency, with participants categorized as consuming alcohol less than once per week or at least once per week. Obesity status was determined using BMI categories: underweight (BMI < 18.5 kg/m^2^), normal weight (BMI ≥ 18.5 to <23 kg/m^2^), overweight (BMI ≥ 23 to <25 kg/m^2^), obese I (BMI ≥ 25 to <30 kg/m^2^), and obese II (BMI ≥ 30 kg/m^2^) [28].

### 2.2. Outcome (Esophageal Cancer)

To minimize the occurrence of false positives, the identification of esophageal cancer cases was based on the specific ICD-10 code for esophageal cancer (C15) when patients had visited the clinics on more than three occasions for diagnostic assessments related to esophageal cancer or if they had visited the clinics at least once and received a confirmed diagnosis of esophageal cancer, followed by subsequent interventions such as surgery, radiotherapy, or chemotherapy [27].

The primary outcome was the odds of esophageal cancer in relation to smoking history, alcohol consumption, and BMI scores.

### 2.3. Covariates

Study participants were organized into 10 age groups, each spanning a 5-year interval. The cohort was further divided into five income categories, ranging from class 1 (lowest income) to class 5 (highest income). Residential regions were initially grouped into 16 categories based on administrative districts and later consolidated into urban and rural areas.

The Charlson Comorbidity Index (CCI), a widely utilized measure of disease burden encompassing 17 potential comorbidities and yielding scores ranging from 0 to 29, was employed in our analysis [29]. However, cases of esophageal cancer were excluded from this calculation to better assess the impact of comorbidities on esophageal cancer development. By excluding comorbidities that are more prevalent in an aging population and those associated with greater disease severity in hospitalized patients, we aimed to minimize potential confounding effects introduced by these factors [29]. We recognized that comorbid conditions could affect the relationship between lifestyle factors and esophageal cancer incidence, leading us to collect and incorporate comorbidity data as covariates in our analysis, thereby addressing and mitigating potential confounding effects [29]. 

### 2.4. Statistical Analysis

To address potential confounding factors and selection bias, we employed propensity score matching. This method aimed to reduce differences between the esophageal cancer and control groups by calculating propensity scores using baseline factors such as age, sex, income, and area of residence [30]. These scores were then used to individually pair esophageal cancer participants with control participants who had similar propensity scores. 

To assess the balance of matched data between the two groups, we examined proportions for categorical variables and calculated means with standard deviations for continuous variables. Any remaining bias was addressed by evaluating absolute standardized differences in covariates before and after matching. A covariate was considered balanced if the absolute standardized difference was ≤0.20 [31]. If a covariate had an absolute standardized difference exceeding 0.20 after matching, we performed additional adjustments using a multivariable logistic regression analysis [31]. 

To analyze the odds ratios (ORs) of smoking history, alcohol consumption, and BMI scores for esophageal cancer, we utilized conditional logistic regression in the matched groups, controlling for age, sex, income, and place of residence. We employed two models: a crude model and an adjusted model that considered age, sex, income, region, and the CCI.

Subgroup analyses were performed by stratifying participants based on age (<55 and ≥55 years) and sex (men and women) using conditional logistic regression, with the division into age groups determined by the median participant age. Additional subgroup analyses categorized participants by smoking status (non-smoker, ex-smoker, and current smoker), alcohol consumption (<1 time per week and ≥1 time per week), and BMI (underweight, normal weight, overweight, obese I, and obese II) using unconditional logistic regression. Statistical analyses were conducted using SAS version 9.4 (SAS Institute Inc., Cary, NC, USA), with statistical significance defined as two-tailed *p*-values of <0.05.

## 3. Results

This study involved 1114 participants with esophageal cancer and 4456 control participants. Given the precise matching of the esophageal cancer and control groups, demographic characteristics such as age group, sex, economic status, and residential region were identical in both groups, indicated by a standardized difference of 0.00 (Table 1).

In the esophageal cancer group, higher proportions were observed for individuals with a CCI score of ≥1 (97.93% vs. 51.21%), current smoking status (37.07% vs. 24.37%), and alcohol consumption at least once a week (58.62% vs. 47.67%), compared with the control group. However, the proportion of patients with a BMI score indicating overweight or higher was lower in the esophageal cancer group than in the control group (45.61% vs. 59.31%).

### 3.1. Relations of Smoking, Alcohol, and Obesity Status with Incident Esophageal Cancer

**Figure 2:**
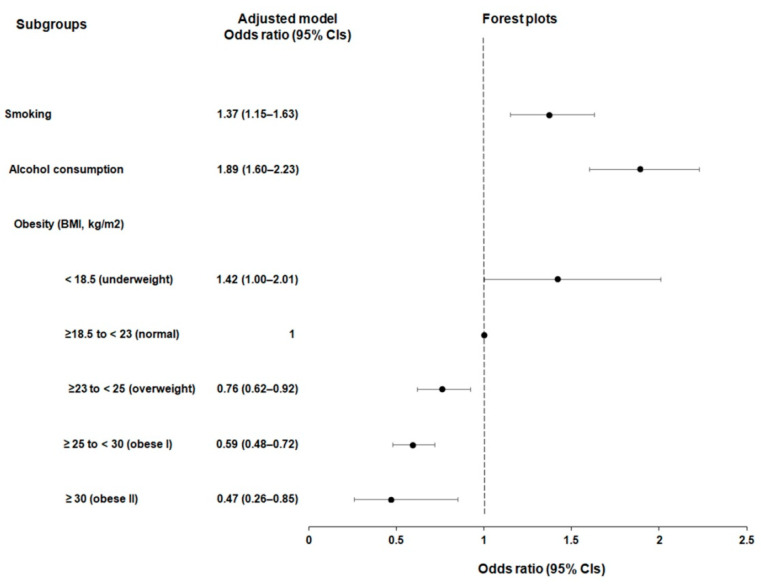
Forest plots illustrating the adjusted odds ratios (aOR) and corresponding 95% confidence intervals (CIs) for lifestyle factors including smoking, alcohol consumption, and obesity status in relation to incident esophageal cancer.

The potential influence of lifestyle factors including smoking status, alcohol consumption, and obesity status on the occurrence of esophageal cancer was analyzed and compared with that in the controls (Table 2; Figure 2).

Smoking and alcohol consumption were associated with increased odds of developing esophageal cancer (OR = 1.37, 95% CI = 1.15–1.63, *p* = 0.001 for smoking, and OR = 1.89, 95% CI = 1.60–2.23, *p* < 0.001 for alcohol consumption). Conversely, higher BMI scores indicating overweight (BMI ≥ 23 to <25 kg/m^2^), obese I (BMI ≥ 25 to <30 kg/m^2^), and obese II (BMI ≥ 30 kg/m^2^) were associated with reduced odds of esophageal cancer ([OR = 0.76, 95% CI = 0.62–0.92, *p* = 0.004] for overweight, [OR = 0.59, 95% CI = 0.48–0.72, *p* < 0.001] for obese I, and [OR = 0.47, 95% CI = 0.26–0.85, *p* = 0.013] for obese II), where we noted a progressive reduction in the magnitudes of esophageal cancer probability in correlation with increasing BMI. Underweight (BMI < 18.5 kg/m^2^) was marginally associated with the incident esophageal cancer (OR = 1.42, 95% CI = 1.00–2.01, *p* = 0.050).

### 3.2. Subgroup Analyses 

**Figure 3:**
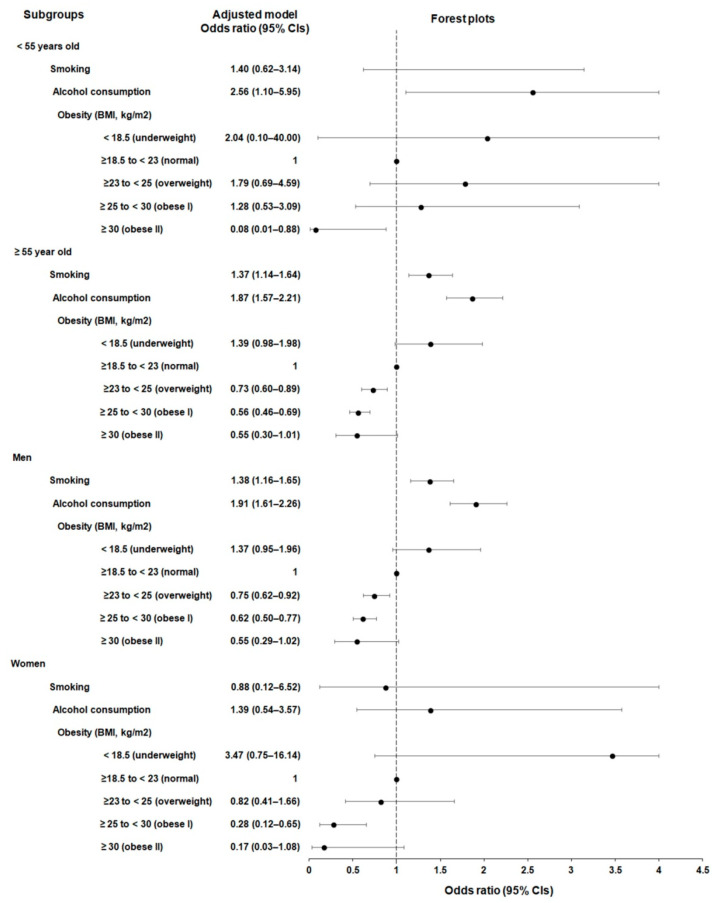
Forest plots for adjusted odd ratios (aOR) (95% confidence intervals, CIs) of smoking, alcohol consumption, and body mass index for esophageal cancer in each stratified group according to age and sex.

Subgroup analyses were performed to investigate the associations of esophageal cancer likelihood within specific subgroups. In the age- and sex-stratified analyses (Table 3; Figure 3), a history of current smoking was linked to a higher likelihood of esophageal cancer in the group aged ≥55 years (aOR = 1.37, 95% CI = 1.14–1.64, *p* = 0.001) and among male individuals (aOR = 1.38, 95% CI = 1.16–1.65, *p* = 0.001).

Similarly, a history of alcohol consumption (≥1 time per week) was associated with an increased probability of esophageal cancer in all age groups (aOR = 2.56, 95% CI = 1.10–5.95, *p* = 0.029 for the <55 years subgroup and aOR = 1.87, 95% CI = 1.57–2.21, *p* < 0.001 for the ≥55 years subgroup) and among men (OR = 1.91, 95% CI = 1.61–2.26, *p* < 0.001). Interestingly, a consistent inverse association was observed between higher BMI scores (indicating overweight or obesity) and the likelihood of esophageal cancer across all age groups and in both sexes.

**Figure 4:**
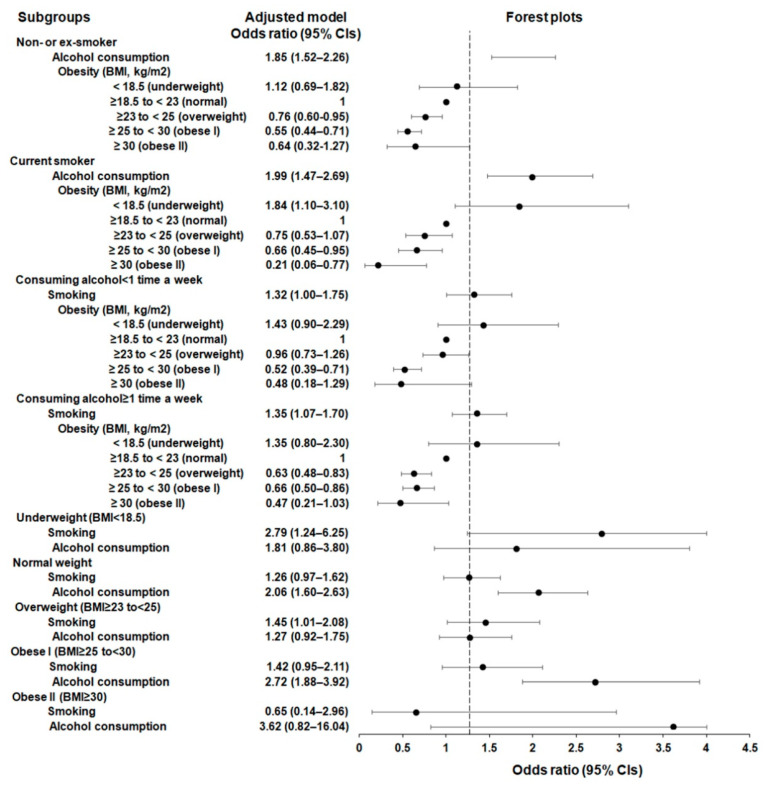
Forest plots for adjusted odd ratios (aORs) (95% confidence intervals, CIs) of smoking, alcohol consumption, and body mass index for esophageal cancer in each group.

Further detailed subgroup analyses were conducted within each smoking, alcohol, and BMI score category (Table 4; Figure 4). For the non-smoker or ex-smoker groups, alcohol consumption showed a positive association with higher odds of esophageal cancer (aOR = 1.85, 95% CI = 1.52–2.26, *p* < 0.001), while being overweight or obese I showed a negative association with the likelihood of esophageal cancer (aOR = 0.76, 95% CI = 0.60–0.95, *p* = 0.018 for overweight and aOR = 0.55, 95% CI = 0.44–0.71, *p* < 0.001 for obese I).

In the current-smoker group, both alcohol consumption (aOR = 1.99, 95% CI = 1.47–2.69, *p* < 0.001) and being underweight (aOR = 1.84, 95% CI = 1.10–3.10, *p* = 0.020) were positively associated with increased likelihoods of esophageal cancer, whereas obese I (BMI ≥ 25 to <30 kg/m^2^; aOR = 0.66, 95% CI = 0.45–0.95, *p* = 0.027) and obese II (BMI ≥ 30 kg/m^2^; aOR = 0.21, 95% CI = 0.06–0.77, *p* = 0.018) were negatively associated with the likelihood of esophageal cancer. 

Among individuals who consumed alcohol at least once a week, smoking and the likelihood of esophageal cancer showed a positive relationship (aOR = 1.35, 95% CI = 1.07–1.70, *p* = 0.011); however, being overweight (aOR = 0.63, 95% CI = 0.48–0.83, *p* = 0.001) or obese I (BMI ≥ 25 to <30 kg/m^2^) (aOR = 0.66, 95% CI = 0.50–0.86, *p* = 0.003) showed an inverse association with the likelihood of esophageal cancer.

Taken together, the subgroup analyses revealed that smoking history was consistently associated with an increased likelihood of esophageal cancer, particularly among older individuals (age ≥ 55 years) and men; a history of alcohol consumption was related to an increased probability of esophageal cancer in all age groups and among men. Among individuals who consumed alcohol at least once a week, there was a positive relationship between smoking and the likelihood of esophageal cancer. However, higher BMI scores (overweight or obesity) were consistently linked to a reduced probability of esophageal cancer across all age groups in both men and women. Consistent inverse relationships between higher BMI scores and the likelihood of esophageal cancer were observed among obese and current smokers, as well as among individuals who consumed alcohol specifically within the overweight and obese I categories. However, underweight individuals and current smokers were more likely to develop esophageal cancer.

## 4. Discussion

This comprehensive nationwide cohort study involved a thorough examination through simultaneous evaluation of various lifestyle factors, such as smoking, alcohol consumption, and BMI status, to ascertain their individual or combined effects on the risk for esophageal cancer. We found that smoking and alcohol consumption were independent risk factors for esophageal cancer, and this positive correlation persisted significantly for smoking and alcohol consumption among men aged >55 years and men in all age groups, respectively. Conversely, higher BMI (overweight and obesity) was associated with a lowered likelihood of esophageal cancer, regardless of age or sex, particularly among smokers or alcohol users. A combined positive influence on the likelihood of esophageal cancer was observed between current smoking and alcohol consumption, whereas a collective negative impact was evident among individuals who were both obese and current smokers, as well as among overweight or obese alcohol drinkers. However, underweight and current smokers (BMI < 18.5 kg/m^2^) displayed an increased propensity for incident esophageal cancer. The results remained significant even after adjusting for potential confounders. Given the modifiable nature of these risk factors, lifestyle modifications could emerge as clinically valuable management strategies for individuals at risk and could potentially mitigate the risk for esophageal cancer. Our findings enable a better understanding of the contributory factors of esophageal cancer by comprehensively and simultaneously evaluating various lifestyle elements, such as smoking, alcohol consumption, and BMI status.

We confirmed that both smoking and alcohol consumption may play separate roles as risk factors for esophageal cancer within the South Korean population. This observation is noteworthy due to the distinctive features of esophageal cancer in South Korea, characterized by its comparatively low incidence and elevated mortality rates [5,6]. Independent smoking or alcohol consumption slightly increased the likelihood of esophageal cancer by 1.37 times (95% CI = 1.15–1.63) and 1.89 times (95% CI = 1.60–2.23), respectively. This notable association was consistently prominent in men aged >55 years for smoking, and in men across all age groups for alcohol consumption. These findings emphasize the sex-specific risks of smoking and alcohol intake for esophageal cancer, with a respective 38% and 91% increase in risk for male individuals, whereas these effects were not significant for the female South Korean population. In South Korea, in 2018, esophageal cancer ranked as the second most impactful factor that contributed to the cancer burden associated with smoking and alcohol intake in men (31.2%), with a notably lower contribution in women (1.7%) [15]. These figures are comparatively lower than the typically observed population attributable fractions of 60–75% for esophageal cancer that are attributed to smoking and alcohol consumption [32,33]. With South Korea’s rapid industrialization and urbanization affecting women’s socioeconomic status, resulting in increased smoking and alcohol consumption among women compared with the past [34,35], sex emerges as a crucial variable in understanding the impact of lifestyle factors on esophageal cancer. Nevertheless, female sex does not seem to have a contributory role in the risk for esophageal cancer. The limited and temporary impact of smoking and alcohol consumption in women could potentially be understood by considering that the highest rates of alcohol consumption and smoking occur within the 19–29 age group, followed by a significant decrease after this [34]. Discontinuing smoking may swiftly and substantially reduce the risk of esophageal cancer, with this decline being closely linked to the duration of smoking cessation [19,36]. Of concern, in individuals aged <55 years, alcohol consumption conferred a 2.56-times higher probability (95% CI = 1.10–5.95) of incident esophageal cancer. This indicates the need for caution in younger age groups in terms of the risk for esophageal cancer. Our findings align with information extracted from 17 cohort studies conducted in Western countries, wherein a lower age at diagnosis (<55 years) amplified the odds associated with alcohol-related risks, particularly in esophageal squamous cell carcinoma [37].

Notably, we identified a combined positive effect between current smoking and alcohol intake (≥1 time a week), which conferred an up to 1.99-fold increased likelihood of esophageal cancer (95% CI 1.47–2.69) and surpassed the individual effects of smoking or alcohol consumption alone. Simultaneous smoking and alcohol consumption were linked to a 3.28-fold increase in esophageal cancer risk based on a meta-analysis [38], although the collective impacts are debatable [14]. The impact of smoking and alcohol consumption on the risk for esophageal cancer in our study seems to closely mirror the findings of previous research in East Asian countries known for their high incidence rates of esophageal cancer, including China (1.33- to 2.06-fold and 2.02-fold increased risk) [12,13,39], Taiwan (2.0-fold and 1.70-fold elevated risk) [40], and Japan (3.73-fold and 3.3-fold increased risk) [8], as well as among Western white populations in population-based studies (1.96- to 2.5-fold and 1.25- to 1.75-fold elevated risk) [19,41,42]. Despite the relatively low incidence of esophageal cancer in South Korea, the comparable impacts, without much difference of smoking and alcohol-associated risks, to those observed in high-incidence countries could indicate that the proportional contribution of alcohol and smoking to the risk of esophageal cancer might be relatively and notably significant. In South Korea, a significant volume of alcohol has been consumed, averaging 10.2 L per year in 2016, which ranked second highest among Asian countries, trailing only Laos (10.4 L) [34]. Of note, the trend in total alcohol consumption in South Korea underwent a 1.8-fold increase from 1998 to 2018 [34]. The recent enforcement of stringent tobacco regulations has significantly reduced the smoking prevalence among adult male individuals (from 47.3% in 2011 to 31.3% in 2021) [43]. However, despite this substantial reduction, the proportion of male smokers still ranks fifth among OECD member nations [43], notably surpassing the rates in Singapore (28.3%), Australia (14.3%), and Hong Kong (10.0%) [33].

In this study, higher BMI scores, specifically overweight and obesity, were linked to a reduced risk for esophageal cancer. Overweight individuals had a 24% lower risk compared with those with a normal BMI, whereas obese individuals exhibited even lower odds, with, respectively, a 41%- and 53%-lower risk for obese I (BMI ≥ 25 to <30 kg/m^2^) and obese II (BMI ≥ 30 kg/m^2^) categories; this suggests a progressive decrease in esophageal cancer risk with an increase in the BMI categories. Similarly, meta-analyses have indicated that the relative risk of esophageal cancer is 0.71 (95% CI = 0.60–0.84) and 0.63 (95% CI = 0.60–0.84) in overweight and obese individuals, respectively [20], with an overall estimated risk of 0.69 (95% CI = 0.63–0.75) [18]. Our findings align with previous studies in Far East Asia, where a significant inverse association between being overweight or obese (BMI ≥ 25 kg/m^2^) and the risk of esophageal cancer has been observed, such as in Japan (aHR 0.59, 95% CI = 0.52–0.67) [44] and China (0.75, 95% CI = 0.64–0.89) [18]. The effect of BMI on esophageal cancer risk in our study seems comparable to that reported in previous meta-analyses that were primarily conducted in Caucasians (0.64, 95% CI = 0.56–0.73 for esophageal squamous cell carcinoma) [41]. 

In this study, BMI data were obtained from the 2 years immediately preceding the index date. A previous study classified subjects according to changes in their BMI between the baseline assessment and health check-ups conducted 2 and 4 years before the diagnosis of esophageal cancer [21]. The study consistently found that weight loss was associated with a reduced likelihood of esophageal cancer for both the 2-year and 4-year check-up periods [21]. It appears that evaluating BMI several years before diagnosis may provide a more relevant correlation between BMI and the likelihood of developing esophageal cancer when compared with BMI data from over a decade ago.

Intriguingly, our study consistently demonstrated an inverse relationship between esophageal cancer incidence and lower BMI among both current smokers and individuals who consumed alcohol at least once a week, regardless of age or sex. This pattern aligns with findings from an analysis of data from 10 population-based cohort studies in Japan, which also revealed a more pronounced inverse association with esophageal cancer among those with a history of smoking (aHR 0.52, 95% CI = 0.46–0.58), as well as in male (0.56, 95% CI = 0.49–0.64) and females individuals (0.74, 95% CI = 0.59–0.94) [44]. However, we noted the heterogeneous impact of BMI on the likelihood of esophageal cancer, particularly in relation to smoking. Although being underweight (BMI < 18.5 kg/m^2^) as an isolated factor demonstrated a statistically marginal association with incident esophageal cancer in our study (1.42, 95% CI = 1.00–2.01, *p* = 0.050), the combined consideration of being underweight (BMI < 18.5 kg/m^2^) and a current smoking revealed a 2.79-fold increase in the probability of esophageal cancer (95% CI = 1.24–6.25), which may indicate the presence of a potentially significant modifying effect between low BMI and smoking on the risk for esophageal cancer [37,44]. Our findings are in line with those from previous consistent research, where individuals who were both underweight and ever-smokers exhibited a 1.60-fold increased risk of esophageal cancer (95% CI = 1.22–2.09), and those who were overweight or obese and ever-smokers experienced a 42% risk reduction (95% CI = 0.48–0.71) [44]. Furthermore, an additional observation highlights the heightened impact of smoking at a lower BMI [37].

The underlying mechanisms whereby smoking, alcohol consumption, and obesity influence esophageal cancer development are complex and multifaceted [45]. The toxic effects of both smoking and alcohol cause genetic and epigenetic alterations, inflammation, oxidative stress, and DNA damage, which can promote carcinogenesis and cancer progression [45,46]. A recent global comparative transcriptome study revealed 19 genes that exhibit unique expression patterns across different populations, suggesting population-specific variations in esophageal cancers [47]. Additionally, toxicogenomic analysis identified tobacco smoking as the predominant risk factor and emphasized a strong genetic–environmental interaction that confers a heightened risk for esophageal cancer [47]. Cigarette smoke primarily induces cancer by forming covalent bonds between its carcinogenic components and DNA, leading to the formation of DNA adducts that cause the mutations in crucial genes, including *FHIT*, that are associated with esophageal cancer. However, nicotine, the main addictive component, triggers EGFR tyrosine kinase activation in oral and esophageal epithelial cells, modifies their behavior to inhibit apoptosis, promotes angiogenesis, modulates inflammation, affects immune responses, and enhances tumor cell invasiveness [46]. In combination with alcohol, cigarette smoking significantly increases acetaldehyde levels in saliva, and individuals with ALDH2 deficiency have a reduced capacity to metabolize this compound [48].

The process of ethanol-induced carcinogenesis is closely tied to liver metabolism. Alcohol dehydrogenase converts ethanol into acetaldehyde, which is then metabolized into acetate by aldehyde dehydrogenase 2 (ALDH2) [45]. This interaction, when associated with biological oxidation, can mitigate membrane lipid peroxidation caused by acetaldehyde, reduce the production of reactive oxygen metabolites stemming from acetaldehyde accumulation, and consequently decrease cellular damage [49]. Independently, the presence of higher concentrations of acetaldehyde in saliva, compared with blood, may constitute toxic alcohol-related acetaldehyde exposure because of the direct contact with the esophageal mucosa [48]. A substantial proportion, approximately 40–50%, of East Asians, including South Koreans [50], exhibit very low ALDH2 activity due to a specific single-nucleotide polymorphism in the coding region of ALDH2 that results in the substitution of lysine with glutamine at position 487, which subsequently increases their vulnerability to the carcinogenic effects of alcohol by elevating acetaldehyde exposure [49]. In genome-wide association studies, these genetic factors of multiple functional variants within *ADH1B* and *ALDH2* genes that lead to heightened acetaldehyde levels after alcohol consumption may interact with environmental factors, particularly tobacco and alcohol use, and result in a significantly heightened risk of esophageal cancer—146.4 to 190 times higher than in individuals without these genetic and lifestyle risk factors [51,52]. In a recent extensive bioinformatics analysis that compared esophageal cancer and normal esophageal tissues, the downregulated genes were found to be primarily associated with pathways related to drug metabolism, chemical carcinogenesis, and xenobiotic metabolism by cytochrome P450, with identified key metabolites, such as acetaldehyde and oxygen, which are linked to esophageal cancer-related genes [53].

The molecular mechanisms underlying the influence of weight status on incident esophageal cancer remain unclear. The potential mechanisms that mediate the relevance between body weight and esophageal cancer have been largely identified in the majority of esophageal adenocarcinomas and include the insulin-like growth factor pathway, adipokines produced by the adipose tissue (adiponectin and leptin), sex hormone disturbances, metabolic alterations, and immune system function [45]. Furthermore, the biological effects of obesity on esophageal squamous cell carcinoma may include the abnormal adipokine secretion, inflammation, oxidative stress, tumor microenvironment, metabolites, immunity, and complex effects [54]. Esophageal squamous cell carcinoma is the predominant form of esophageal cancer in South Korea [5]; thus, our data on esophageal cancer were assumed to be mainly drawn from esophageal squamous cell carcinoma. The association of being underweight with an increased likelihood of esophageal cancer in our study may be because severe weight loss may weaken the immune system and easily exacerbate esophageal cancer pathogenesis [3]. In recent gene enrichment and pathway analysis, downregulated differential expression genes in esophageal squamous cell carcinomas that mainly participated in the biological processes have been associated with neutrophil activation in the immune response, neutrophil degranulation, and neutrophil-medicated immunity [53]. This indicates the immune dysfunction in esophageal cancer. The downregulated differential expression genes were predominantly associated with functions endopeptidase inhibitor activity, serine-type endopeptidase inhibitor activity, cadherin binding, and steroid hydroxylase activity [53], which might be linked with the inhibition of tumor cell invasion, migration, and metastasis [55], as well as cancer lipid metabolism, including in esophageal squamous cell carcinoma [56], which might explain the possible link between a high BMI and the decreased probability of esophageal cancer in the present study. Nevertheless, a recent Mendelian randomization and genome-wide association study found no evidence to support a causal association between BMI and risk of esophageal cancer in an Asian population [57].

Notably, this study stands out due to its large sample size of esophageal cancer patients and control participants, utilization of a nationwide representative cohort population using a propensity score matching approach to ensure balanced distributions of baseline characteristics, and simultaneous analysis of multiple lifestyle factors including smoking, alcohol consumption, and obesity status. The use of a large cohort population facilitated the random selection and matching of the comparison group with esophageal cancer patients, reducing the potential bias in the selection process and mimicking the rigor of randomized trials. Despite the typical occurrence of esophageal cancer in older individuals, our study, which included 1114 esophageal cancer cases matched 1:4 with 4456 control participants within the relevant age groups, achieved a balanced distribution of sex and age in the cohort. Demographic heterogeneity among participants might have affected the strength of associations observed in the original research groups [22]. Consequently, through this approach, we have established potential links between smoking, alcohol consumption, and BMI scores and the likelihood of developing esophageal cancer. Since the KNHIS-HSC data encompassed every hospital and clinic nationwide without exceptions, no medical history data were lost during follow-up, which may indicate the generalizability and reliability of our data.

Nonetheless, there are some limitations to consider. First, the health insurance dataset did not include information on *Helicobacter pylori* infection, cancer stage, histological characteristics, differentiation status, family medical history, genetic predisposition, and various other factors such as dietary habits, physical activity, and specific types of alcohol. These omissions have the potential to introduce confounding effects and restrict the comprehensiveness of the study. In South Korea, alcohol consumption is predominantly characterized by the consumption of Soju, a traditional South Korean alcoholic beverage, and beer [34]. Although we were unable to consider different types of alcohol due to a lack of data, it is important to note that the specific kinds of alcoholic beverages consumed and their respective alcohol or acetaldehyde concentrations may influence the risk of esophageal cancer. For instance, the consumption of Calvados, an apple brandy with higher acetaldehyde exposure, explained a significant portion of the increased esophageal cancer risk in certain French regions [58], while a Chinese study suggested a stronger association with esophageal cancer linked to spirits consumption, possibly due to their higher ethanol concentration compared with rice wine or beer [12]. Second, since this study recruited participants based on diagnosis codes and exclusively involved South Korean subjects, it was not possible to completely eradicate unmeasured confounding effects. The data sourced from South Korea may not fully capture the characteristics of other Asian populations, given the significant geographic variations in the incidence and relative prevalence of esophageal cancer across Asia. Third, self-reported data of smoking and alcohol drinking may introduce recall bias. Participants may provide inaccurate or inconsistent information regarding their habits, potentially influencing the precision of the associations between these factors and the development of esophageal cancer. Finally, the retrospective case–control study design does not allow for the determination of causality based on the current data. Instead, it can only reveal associations and is susceptible to recall bias and selection bias. Regarding obesity, this study did not consider other obesity indicators such as waist-to-hip ratio and body fat percentage. Therefore, it is important to acknowledge certain limitations in the findings, including the absence of comprehensive variables, geographic specificity, recall bias, and the retrospective design. These limitations should be considered when formulating prevention and intervention strategies for esophageal cancer, both within the South Korean population and in broader contexts.

## 5. Conclusions

This study may offer further support for the connections between smoking, alcohol consumption, and obesity status, and their respective impacts on the risk of esophageal cancer within the South Korean population. We found that smoking and alcohol consumption were independent risk factors for esophageal cancer, and the proportional influence of alcohol and smoking on esophageal cancer risk remains significant, even in South Korea with a relatively low incidence of the disease. This positive correlation persisted significantly for smoking and alcohol consumption among men aged >55 years and men in all age groups, respectively. Female sex does not seem to independently contribute to the risk of esophageal cancer. Conversely, overweight and obesity status were associated with a lowered likelihood of esophageal cancer, regardless of age or sex, particularly among smokers or alcohol users. Certain effect modifications likely exist in current smokers who are underweight, with a resultant enhanced likelihood of esophageal cancer. Overall, our findings may indicate the nuanced associations between lifestyle factors (such as alcohol consumption, smoking, and BMI) and the likelihood of esophageal cancer within specific subgroups in the South Korean population.

## Figures and Tables

**Table 1:** General characteristics of the participants.

Characteristics		Total Participants		
		Esophageal Cancer	Control	Standardized Difference
Age (years; *n*, %)				0.00
	40–44	2 (0.18)	8 (0.18)	
	45–49	15 (1.35)	60 (1.35)	
	50–54	62 (5.57)	248 (5.57)	
	55–59	131 (11.76)	524 (11.76)	
	60–64	187 (16.79)	748 (16.79)	
	65–69	239 (21.45)	956 (21.45)	
	70–74	210 (18.85)	840 (18.85)	
	75–79	164 (14.72)	656 (14.72)	
	80–84	87 (7.81)	348 (7.81)	
	85+	17 (1.53)	68 (1.53)	
Sex (*n*, %)				0.00
	Male	1035 (92.91)	4140 (92.91)	
	Female	79 (7.09)	316 (7.09)	
Income (*n*, %)				0.00
	1 (lowest)	190 (17.06)	760 (17.06)	
	2	142 (12.75)	568 (12.75)	
	3	195 (17.50)	780 (17.50)	
	4	235 (21.10)	940 (21.10)	
	5 (highest)	352 (31.60)	1408 (31.60)	
Region of residence (*n*, %)				0.00
	Urban	420 (37.70)	1680 (37.70)	
	Rural	694 (62.30)	2776 (62.30)	
CCI score (*n*, %)				1.81
	0	23 (2.06)	2174 (48.79)	
	1	24 (2.15)	826 (18.54)	
	2	300 (26.93)	534 (11.98)	
	3	214 (19.21)	382 (8.57)	
	≥4	553 (49.64)	540 (12.12)	
Obesity status (BMI, kg/m^2^)				0.31
	<18.5 (underweight)	78 (7.00)	156 (3.50)	
	≥18.5 to <23 (normal)	528 (47.40)	1657 (37.19)	
	≥23 to <25 (overweight)	270 (24.24)	1213 (27.22)	
	≥25 to <30 (obese I)	220 (19.75)	1335 (29.96)	
	≥30 (obese II)	18 (1.62)	95 (2.13)	
Smoking status (*n*, %)				0.28
	Non-smoker or ex-smoker	701 (62.93)	3370 (75.63)	
	Current smoker	413 (37.07)	1086 (24.37)	
Alcohol consumption (*n*, %)				0.22
	<1 time a week	461 (41.38)	2332 (52.33)	
	≥1 time a week	653 (58.62)	2124 (47.67)	

Abbreviations: BMI, body mass index; CCI, Charlson Comorbidity Index.

**Table 2:** Crude and adjusted odd ratios (95% confidence intervals) of smoking, alcohol consumption, and obesity status for esophageal cancer.

	N of Esophageal Cancer (Exposure/Total, %)	N of Control (Exposure/Total, %)	Odd Ratios for Esophageal Cancer (95% Confidence Interval)			
			Crude †	*p*	Adjusted †‡	*p*
Smoking status	413/1114 (37.07)	1086/4456 (24.37)	1.92 (1.66–2.23)	<0.001 *	1.37 (1.15–1.63)	0.001 *
Alcohol consumption	653/1114 (58.62)	2124/4456 (47.67)	1.60 (1.40–1.84)	<0.001 *	1.89 (1.60–2.23)	<0.001 *
Obesity status (BMI, kg/m^2^)						
<18.5 (underweight)	78/1114 (7.00)	156/4456 (3.50)	1.59 (1.19–2.12)	0.002 *	1.42 (1.00–2.01)	0.050
≥18.5 to <23 (normal)	528/1114 (47.40)	1657/4456 (37.19)	1.00		1.00	
≥23 to <25 (overweight)	270/1114 (24.24)	1213/4456 (27.22)	0.69 (0.59–0.82)	<0.001 *	0.76 (0.62–0.92)	0.004 *
≥25 to <30 (obese I)	220/1114 (19.75)	1335/4456 (29.96)	0.51 (0.43–0.61)	<0.001 *	0.59 (0.48–0.72)	<0.001 *
≥30 (obese II)	18/1114 (1.62)	95/4456 (2.13)	0.59 (0.35–0.98)	0.041	0.47 (0.26–0.85)	0.013 *

Abbreviation: BMI, body mass index. * Conditional logistic regression analysis, significance at *p* < 0.05. † Stratified model for age, sex, income, and region of residence. ‡ Adjusted model for Charlson Comorbidity Index, obesity, smoking state (current smoker compared with non-smoker or ex-smoker), and frequency of alcohol consumption (≥1 time a week compared with <1 time a week).

**Table 3:** Crude and adjusted odd ratios (95% confidence intervals) of smoking, alcohol consumption, and obesity status for esophageal cancer in each stratified group according to age and sex.

Characteristics	N of Esophageal Cancer	N of Control	ORs of Esophageal Cancer			
	(Exposure/Total, %)	(Exposure/Total, %)	Crude †	*p*	Adjusted †‡	*p*
<55 years old (*n* = 395)						
Smoking	42/79 (53.2)	119/316 (37.7)	2.04 (1.20–3.48)	0.009 *	1.40 (0.62–3.14)	0.421
Alcohol consumption	55/79 (69.6)	163/316 (51.6)	2.38 (1.34–4.20)	0.003 *	2.56 (1.10–5.95)	0.029 *
Obesity status (BMI, kg/m^2^)						
<18.5 (underweight)	3/79 (3.8)	4/316 (1.3)	2.40 (0.51–11.42)	0.271	2.04 (0.10–40.00)	0.640
≥18.5 to <23 (normal)	31/79 (39.2)	102/316 (32.3)	1.00		1.00	
≥23 to <25 (overweight)	19/79 (24.1)	80/316 (25.3)	0.78 (0.41–1.47)	0.438	1.79 (0.69–4.59)	0.229
≥25 to <30 (obese I)	25/79 (31.7)	121/316 (38.3)	0.67 (0.37–1.22)	0.191	1.28 (0.53–3.09)	0.586
≥30 (obese II)	1/79 (1.3)	9/316 (2.9)	0.37 (0.05–3.02)	0.353	0.08 (0.01–0.88)	0.039 *
≥55 years old (*n* = 5175)						
Smoking	371/1035 (35.9)	967/4140 (23.4)	1.92 (1.65–2.23)	<0.001 *	1.37 (1.14–1.64)	0.001 *
Alcohol consumption	598/1035 (57.8)	1961/4140 (47.4)	1.56 (1.36–1.80)	<0.001 *	1.87 (1.57–2.21)	<0.001 *
Obesity status (BMI, kg/m^2^)						
<18.5 (underweight)	75/1035 (7.3)	152/4140 (3.7)	1.56 (1.16–2.09)	0.003 *	1.39 (0.98–1.98)	0.066
≥18.5 to <23 (normal)	497/1035 (48)	1555/4140 (37.6)	1.00		1.00	
≥23 to <25 (overweight)	251/1035 (24.3)	1133/4140 (27.4)	0.69 (0.58–0.81)	<0.001 *	0.73 (0.60–0.89)	0.002 *
≥25 to <30 (obese I)	195/1035 (18.8)	1214/4140 (29.3)	0.50 (0.41–0.60)	<0.001 *	0.56 (0.46–0.69)	<0.001 *
≥30 (obese II)	17/1035 (1.6)	86/4140 (2.1)	0.61 (0.36–1.04)	0.069	0.55 (0.30–1.01)	0.052
Men (*n* = 5175)						
Smoking	410/1035 (39.6)	1079/4140 (26.1)	1.93 (1.66–2.23)	<0.001 *	1.38 (1.16–1.65)	0.001 *
Alcohol consumption	639/1035 (61.7)	2080/4140 (50.2)	1.61 (1.40–1.86)	<0.001 *	1.91 (1.61–2.26)	<0.001 *
Obesity status (BMI, kg/m^2^)						
<18.5 (underweight)	73/1035 (7.1)	149/4140 (3.6)	1.56 (1.16–2.10)	0.004 *	1.37 (0.95–1.96)	0.088
≥18.5 to <23 (normal)	488/1035 (47.2)	1535/4140 (37.1)	1.00		1.00	
≥23 to <25 (overweight)	248/1035 (24)	1131/4140 (27.3)	0.68 (0.57–0.81)	<0.001 *	0.75 (0.62–0.92)	0.006 *
≥25 to <30 (obese I)	210/1035 (20.3)	1242/4140 (30)	0.52 (0.44–0.63)	<0.001 *	0.62 (0.50–0.77)	<0.001 *
≥30 (obese II)	16/1035 (1.6)	83/4140 (2)	0.60 (0.35–1.03)	0.062	0.55 (0.29–1.02)	0.058
Women (*n* = 395)						
Smoking	3/79 (3.8)	7/316 (2.2)	1.74 (0.44–6.87)	0.430	0.88 (0.12–6.52)	0.898
Alcohol consumption	14/79 (17.7)	44/316 (13.9)	1.39 (0.68–2.81)	0.365	1.39 (0.54–3.57)	0.495
Obesity status (BMI, kg/m^2^)						
<18.5 (underweight)	5/79 (6.3)	7/316 (2.2)	2.13 (0.63–7.15)	0.223	3.47 (0.75–16.14)	0.112
≥18.5 to <23 (normal)	40/79 (50.6)	122/316 (38.6)	1.00		1.00	
≥23 to <25 (overweight)	22/79 (27.9)	82/316 (26)	0.81 (0.45–1.47)	0.495	0.82 (0.41–1.66)	0.584
≥25 to <30 (obese I)	10/79 (12.7)	93/316 (29.4)	0.34 (0.16–0.71)	0.004 *	0.28 (0.12–0.65)	0.004 *
≥30 (obese II)	2/79 (2.5)	12/316 (3.8)	0.52 (0.11–2.46)	0.412	0.17 (0.03–1.08)	0.060

Abbreviations: BMI, body mass index; OR, odds ratio. * Conditional logistic regression analysis, significance at *p* < 0.05. † Stratified model for age, sex, income, and region of residence. ‡ Adjusted model for Charlson Comorbidity Index, obesity, smoking state (current smoker compared with non-smoker or ex-smoker), and frequency of alcohol consumption (≥1 time a week compared with <1 time a week).

**Table 4:** Crude and adjusted odd ratios (95% confidence intervals) of smoking, alcohol consumption, and obesity status for esophageal cancer in each group.

Characteristics	N of Esophageal Cancer	N of Control	ORs of Esophageal Cancer			
	(Exposure/Total, %)	(Exposure/Total, %)	Crude	*p*	Adjusted †	*p*
Non- or ex-smoker (*n* = 4071)						
Alcohol consumption	353/701 (50.4)	1461/3370 (43.4)	1.33 (1.13–1.56)	0.001 *	1.85 (1.52–2.26)	<0.001 *
Obesity status (BMI, kg/m^2^)						
<18.5 (underweight)	34/701 (4.9)	100/3370 (3.0)	1.30 (0.87–1.96)	0.205	1.12 (0.69–1.82)	0.653
≥18.5 to <23 (normal)	308/701 (43.9)	1180/3370 (35.0)	1.00		1.00	
≥23 to <25 (overweight)	189/701 (27.0)	950/3370 (28.2)	0.76 (0.62–0.93)	0.008	0.76 (0.60–0.95)	0.018 *
≥25 to <30 (obese I)	156/701 (22.3)	1074/3370 (31.9)	0.56 (0.45–0.69)	<0.001 *	0.55 (0.44–0.71)	<0.001 *
≥30 (obese II)	14/701 (2.0)	66/3370 (2.0)	0.81 (0.45–1.47)	0.491	0.64 (0.32–1.27)	0.200
Current smoker (*n* = 1499)						
Alcohol consumption	300/413 (72.6)	663/1086 (61.1)	1.69 (1.32–2.17)	<0.001 *	1.99 (1.47–2.69)	<0.001 *
Obesity status (BMI, kg/m^2^)						
<18.5 (underweight)	44/413 (10.7)	56/1086 (5.2)	1.70 (1.11–2.61)	0.014 *	1.84 (1.10–3.10)	0.020 *
≥18.5 to <23 (normal)	220/413 (53.3)	477/1086 (43.9)	1.00		1.00	
≥23 to <25 (overweight)	81/413 (19.6)	263/1086 (24.2)	0.67 (0.50–0.90)	0.008 *	0.75 (0.53–1.07)	0.112
≥25 to <30 (obese I)	64/413 (15.5)	261/1086 (24.0)	0.53 (0.39–0.73)	<0.001 *	0.66 (0.45–0.95)	0.027 *
≥30 (obese II)	4/413 (1.0)	29/1086 (2.7)	0.30 (0.10–0.86)	0.025 *	0.21 (0.06–0.77)	0.018 *
Consuming alcohol < 1 time a week (*n* = 2793)						
Smoking	113/461 (24.5)	423/2332 (18.1)	1.47 (1.16–1.86)	0.002 *	1.32 (1.00–1.75)	0.051
Obesity status (BMI, kg/m^2^)						
<18.5 (underweight)	34/461 (7.4)	96/2332 (4.1)	1.53 (1.01–2.33)	0.047 *	1.43 (0.90–2.29)	0.134
≥18.5 to <23 (normal)	205/461 (44.5)	886/2332 (38.0)	1.00		1.00	
≥23 to <25 (overweight)	134/461 (29.1)	596/2332 (25.6)	0.97 (0.76–1.24)	0.816	0.96 (0.73–1.26)	0.771
≥25 to <30 (obese I)	83/461 (18.0)	710/2332 (30.5)	0.51 (0.38–0.66)	<0.001 *	0.52 (0.39–0.71)	0.816
≥30 (obese II)	5/461 (1.1)	44/2332 (1.9)	0.49 (0.19–1.25)	0.137	0.48 (0.18–1.29)	0.143
Consuming alcohol ≥ 1 time a week (*n* = 2777)						
Smoking	300/653 (45.9)	663/2124 (31.2)	1.87 (1.57–2.24)	<0.001 *	1.35 (1.07–1.70)	0.011 *
Obesity status (BMI, kg/m^2^)						
<18.5 (underweight)	44/653 (6.7)	60/2124 (2.8)	1.75 (1.16–2.64)	0.008 *	1.35 (0.80–2.30)	0.265
≥18.5 to <23 (normal)	323/653 (49.5)	771/2124 (36.3)	1.00		1.00	
≥23 to <25 (overweight)	136/653 (20.8)	617/2124 (29.1)	0.53 (0.42–0.66)	<0.001 *	0.63 (0.48–0.83)	0.001 *
≥25 to <30 (obese I)	137/653 (21.0)	625/2124 (29.4)	0.52 (0.42–0.66)	<0.001 *	0.66 (0.50–0.86)	0.003 *
≥30 (obese II)	13/653 (2.0)	51/2124 (2.4)	0.61 (0.33–1.13)	0.118	0.47 (0.21–1.03)	0.058
Underweight (BMI < 18.5 kg/m^2^, *n* = 234)						
Smoking	44/78 (56.4)	56/156 (35.9)	2.31 (1.33–4.02)	0.003 *	2.79 (1.24–6.25)	0.013 *
Alcohol consumption	44/78 (56.4)	60/156 (38.5)	2.07 (1.19–3.60)	0.010 *	1.81 (0.86–3.80)	0.119
Normal weight (BMI ≥ 18.5 to <23 kg/m^2^, *n* = 2185)						
Smoking	220/528 (41.7)	477/1657 (28.8)	1.77 (1.44–2.17)	<0.001 *	1.26 (0.97–1.62)	0.081
Alcohol consumption	323/528 (61.2)	771/1657 (46.5)	1.81 (1.48–2.21)	<0.001 *	2.06 (1.60–2.63)	<0.001 *
Overweight (BMI ≥ 23 to <25 kg/m^2^, *n* = 1483)						
Smoking	81/270 (30.0)	263/1213 (21.7)	1.55 (1.15–2.08)	0.004 *	1.45 (1.01–2.08)	0.042 *
Alcohol consumption	136/270 (50.4)	617/1213 (50.9)	0.98 (0.75–1.28)	0.883	1.27 (0.92–1.75)	0.148
Obese I (BMI ≥ 25 to <30 kg/m^2^, *n* = 1555)						
Smoking	64/220 (29.1)	261/1335 (19.6)	1.69 (1.23–2.33)	0.001 *	1.42 (0.95–2.11)	0.087
Alcohol consumption	137/220 (62.3)	625/1335 (46.8)	1.88 (1.40–2.51)	<0.001 *	2.72 (1.88–3.92)	<0.001 *
Obese II (BMI ≥ 30 kg/m^2^, *n* = 113)						
Smoking	4/18 (22.2)	29/95 (30.5)	0.65 (0.20–2.15)	0.480	0.65 (0.14–2.96)	0.579
Alcohol consumption	13/18 (72.2)	51/95 (53.7)	2.24 (0.74–6.79)	0.153	3.62 (0.82–16.04)	0.090

Abbreviations: OR, odds ratio; BMI, body mass index. * Unconditional logistic regression analysis, significance at *p* < 0.05. † Adjusted model for age, sex, income, region of residence, Charlson Comorbidity Index, obesity, smoking state (current smoker compared with non-smoker or ex-smoker), and frequency of alcohol consumption (≥1 time a week compared with <1 time a week).

## Data Availability

All data are available from the database of National Health Insurance Sharing Service (NHISS) https://nhiss.nhis.or.kr/ (accessed on 1 May 2022). The NHISS allows access to all of this data for the any researcher who promises to follow the research ethics at some processing charge. If you want to access the data of this article, you can download it from the website after promising to follow the research ethics.
